# Dopamine Suppresses Osteogenic Differentiation of Rat Bone Marrow-Derived Mesenchymal Stem Cells via AKT/GSK-3*β*/*β*-Catenin Signaling Pathway

**DOI:** 10.1155/2022/4154440

**Published:** 2022-06-29

**Authors:** Zhili Kuang, Zheng Chen, Shaoqin Tu, Zhihui Mai, Lin Chen, Xiaoning Kang, Xiaochuan Chen, Jiaming Wei, Yuxuan Wang, Yun Peng, Hong Ai

**Affiliations:** ^1^Department of Stomatology, The Third Affiliated Hospital of Sun Yat-sen University, Guangzhou, China; ^2^Department of Stomatology, Huazhong University of Science and Technology Union Shenzhen Hospital (Nanshan Hospital), Shenzhen, China; ^3^Guangdong Provincial Key Laboratory of Stomatology, Guanghua School of Stomatology, Hospital of Stomatology, Sun Yat-sen University, Guangzhou, China

## Abstract

Nervous system is critically involved in bone homeostasis and osteogenesis. Dopamine, a pivotal neurotransmitter, plays a crucial role in sympathetic regulation, hormone secretion, immune activation, and blood pressure regulation. However, the role of dopamine on osteogenic differentiation of rat bone marrow-derived mesenchymal stem cells (rBMSCs) remains poorly understood. In this study, we firstly investigated the effect of dopamine on the apoptosis, proliferation, and osteogenic differentiation of rBMSCs. Dopamine did not, however, interfere with the apoptosis and proliferation of rBMSCs. Interestingly, dopamine suppressed the osteogenic differentiation of rBMSCs, as characterized by reduced ALP staining, ALP activity, mineralized nodule formation, and the mRNA and protein levels of osteogenesis-related genes (*Col1a1*, *Alp*, *Runx2*, *Opn*, and *Ocn*). Furthermore, dopamine inactivated AKT/GSK-3*β*/*β*-catenin signaling pathway. Treatment of LiCl (GSK-3*β* inhibitor) rescued the inhibitory effects of dopamine on osteogenic differentiation of rBMSCs. LY294002 (AKT inhibitor) administration exacerbated the inhibitory effects of dopamine on osteogenic differentiation of rBMSCs. Taken together, these findings indicate that dopamine suppresses osteogenic differentiation of rBMSCs via AKT/GSK-3*β*/*β*-catenin signaling pathway. Our study provides new insights into the role of neurotransmitters in bone homeostasis.

## 1. Introduction

Osteoporosis is a systemic skeletal disease characterized by a reduction in bone mineral density and deterioration of bone structure, leading to an increased risk of bone fragility and fractures [1]. Osteoporosis has become a prominent threat to public health in the context of ageing [2]. Currently, osteoporosis is mainly associated with abnormal bone remodeling [3].

Bone remodeling is a dynamic, physiological, and continuous process coupling osteoblastic bone formation and osteoclastic bone resorption [4]. This process is tightly regulated by paracrine and autocrine signals to ensure calcium/phosphate homeostasis and thus maintain a healthy mineralized skeleton. An imbalance between bone formation and bone resorption during bone remodeling leads to bone metabolic diseases such as osteoporosis, osteopetrosis, and osteopenia [5]. Recent studies have suggested that the central and peripheral nervous systems could directly regulate bone remodeling [6–8].

Dopamine (DA), a pivotal catecholamine neurotransmitter of the central nervous system, plays crucial roles in cognition, emotion, memory, attention, vision, reward, sympathetic regulation, and hormonal regulation [9]. Abnormal DA levels are related to neurological and mental disorders such as Parkinson's disease (PD) and Schizophrenia (SZ). Studies have shown that patients with PD or SZ had a higher prevalence of osteoporosis compared to their healthy controls [10–12]. These findings raise the possibility that DA might play a role in bone homeostasis. It was found that mice with genetic deletion of the dopamine transporter (DAT), which mediates DA reuptake from the synapse back into the presynaptic terminals, exhibited reduced bone mass [13]. Previous studies have reported that DA was involved in the osteogenic differentiation of bone marrow-derived mesenchymal stem cells (BMSCs) [14–17].

Currently, Wnt/*β*-catenin signaling pathway is considered the master regulator involved in osteogenesis [18]. Wnt signaling stabilizes cytoplasmic *β*-catenin, induces nucleus translocation of accumulated *β*-catenin, and thus promotes osteogenic differentiation. *β*-Catenin can be regulated by glycogen synthase kinase 3*β* (GSK-3*β*), which acts as a negative regulator of Wnt/*β*-catenin signaling pathway and is implicated in governing osteogenic differentiation. In addition, activation of AKT (phosphorylated-AKT, (p)-AKT) can trigger GSK-3*β* phosphorylation at Ser9, which leads to the inactivation of GSK-3*β*, and then inhibit the degradation of *β*-catenin [19–21].

However, to the best of our knowledge, the role of AKT/GSK-3*β*/*β*-catenin signaling pathway in DA-induced osteogenic differentiation remains to be elucidated. Thus, in this study, firstly we isolated and identified rat bone mesenchymal stem cells (rBMSCs). Then, we evaluated the effect of DA on the apoptosis and proliferation of rBMSCs. Next, we carried out ALP staining, ALP activity assay, Alizarin Red staining, and mRNA and protein levels of genes related to osteogenic differentiation to investigate the effect of DA on osteogenic differentiation. Finally, we used GSK-3*β* inhibitor lithium chloride (LiCl) and AKT inhibitor LY294002 to explore the mechanism by which DA regulated osteogenesis. This study may provide new insights into the mechanisms underlying bone remodeling and a theoretical basis for the treatment of bone defects or osteoporosis.

## 2. Materials and Methods

### 2.1. Isolation, Culture, and Characterization of rBMSCs

The isolation and culture of rBMSCs from the tibias and femurs of 3-week-old male Sprague-Dawley rats were performed as described previously [22, 23]. Briefly, rats were sacrificed by cervical dislocation and sterilized with 75% ethanol. After dissecting the epiphyses of femurs and tibias, the bone marrow cells were flushed out using *α*-minimum essential medium (*α*-MEM, Gibco, USA) and centrifuged at 1000 rpm for 5 min. The cells were resuspended in growth medium containing 10% fetal bovine serum (FBS, Gibco, USA) and 1% Penicillin/Streptomycin (Gibco, USA) and cultured at 37°C in a 5% CO_2_ atmosphere. After 24 hours, the medium was replaced to remove the nonadherent cells and then was completely replaced every 2-3 days. Upon reaching 70–80% confluence, cells were trypsinized and passaged. Cells at passages 3-4 were used for subsequent experiments.

To investigate the multipotency of rBMSCs, the cells were induced to differentiate in osteogenic, adipogenic, or chondrogenic differentiation medium. For osteogenic differentiation, rBMSCs were cultured with osteogenic differentiation medium (OM), which is a growth medium supplemented with 10 mM *β*-glycerophosphate (Sigma, USA), 50 *μ*g/ml ascorbic acid-2-phosphate (Sigma, USA), and 100 nM dexamethasone (Sigma, USA). Adipogenic or chondrogenic differentiation medium was purchased from Cyagen Biosciences. The expression of cell surface markers, including CD29, CD34, CD44, CD45, and CD90 (Cyagen, China) on rBMSCs was identified by flow cytometry. Fluorescein isothiocyanate- (FITC-) conjugated mouse IgG (Invitrogen, USA) was used as a negative control.

### 2.2. Annexin V/PI Staining Assay

Apoptosis of rBMSCs was determined by using FITC-conjugated Annexin V and propidium iodide (PI) Apoptosis Detection kit (Yeasen, China) as per the manufacturer's instructions. Briefly, cells were seeded in 6-well plates at 10^5^ cells/well. After 24 h incubation, cultured cells were exposed to DA (Sigma, USA) at concentrations of 0, 10^−9^, 10^−7^, and 10^−5^ M for 2 days. Then, both floating and adherent cells were harvested and washed twice with precold PBS. The cells were centrifuged and resuspended in 100 *μ*L of binding buffer containing 5 *μ*L Annexin V-FITC and 10 *μ*L PI. After incubation at room temperature in the dark for 15 min and adding 400 *μ*L binding buffer, the mixture was analyzed by flow cytometry within 1 h.

### 2.3. Cell Counting Kit-8 (CCK-8) Assay

The toxicity of DA on rBMSCs was detected by the CCK-8 assay. Briefly, cells were seeded in 96-well plates at 3 × 10^3^ cells/well. After 24 h incubation, cells were treated with dopamine at concentrations of 0, 10^−9^, 10^−7^, and 10^−5^ M for 1, 3, 5, and 7 days, respectively. The CCK-8 assays were performed using the CCK-8 Kit (Dojindo, Japan) according to the manufacturer's protocol.

### 2.4. 5-Ethynyl-2′-Deoxyuridine (EdU) Assay

EdU assay was conducted by using EdU assay Kit (Yeasen, China) according to the manufacturer's instructions. In brief, cells were incubated in growth medium containing EdU for 2 h. Then, the cells were fixed in 4% formaldehyde (Biosharp, China) for 20 min and permeabilized with 0.3% TritonX-100 (Sigma, USA) for 15 min. Subsequently, cells were incubated at room temperature for 30 min in the click reaction cocktail. Cell nuclei were stained by Hoechst 33342 for 30 min. Images were captured under confocal microscopy (Carl Zeiss, Germany).

### 2.5. Alkaline Phosphatase (ALP) Staining

After 7 days' osteogenic induction, ALP staining was performed according to the manufacturer's instructions (Beyotime, China). Cells were washed twice with PBS, fixed with 4% paraformaldehyde for 20 min, rinsed twice with PBS, and then stained with ALP staining for 10 min. Images were captured under an inverted microscope (Carl Zeiss, Germany).

### 2.6. Alkaline Phosphatase (ALP) Activity Assay

Cells were cultured in osteogenic differentiation medium for 7 days. For determination of ALP activity, cells were washed with PBS and assayed for ALP activity with an ALP Assay Kit (Nanjing Jiancheng, China) according to the manufacturer's recommendations. Total protein concentration was measured by using a BCA protein assay kit (Yeasen, China). The ALP activity was expressed as nmol phenol/15 min/mg protein.

### 2.7. Alizarin Red Staining

After the induction of osteogenic differentiation, mineral deposition was detected by Alizarin Red staining (ARS) (Cyagen, China) on day 14. Cells were washed twice with PBS, fixed with 4% paraformaldehyde for 20 min, rinsed twice with PBS, and then stained with Alizarin Red staining for 5 min. Images were captured under an inverted microscope (Carl Zeiss, Germany). For the quantification analysis, the calcium deposits were desorbed with 10% (w/v) cetylpyridinium chloride (Sigma, USA), and the absorbance at 562 nm was measured.

### 2.8. Oil Red “O” Staining

After 14 days of adipogenic induction, lipid droplets were stained with Oil Red “O” (Cyagen, China). Cells were washed twice with PBS, fixed with 4% paraformaldehyde for 20 min, rinsed twice with PBS, and then stained with Oil Red “O” for 30 min. Images were captured under an inverted microscope (Carl Zeiss, Germany).

### 2.9. Quantitative Real-Time Reverse Transcription Polymerase Chain Reaction (qRT-PCR)

Total RNA was extracted by using RNA-Quick Purification Kit (Esunbio, China) as per the manufacturer's protocol. cDNA was synthesized from 1 *μ*g of total RNA with the PrimeScript RT Reagent Kit (Takara, Japan). Real-time PCR assays for collagen type I alpha 1 chain (*Col1a1*), alkaline phosphatase (*Alp*), runt-related transcription factor 2 (*Runx2*), osteopontin (*Opn*), osteocalcin (*Ocn*), adiponectin (*Adipoq*), CCAAT/enhancer-binding protein alpha (*C/ebpα*), fatty acid-binding protein 4 (*Fabp4*), peroxisome proliferator-activated receptor gamma (*Pparγ*), and perilipin 1 (*Plin1*) were performed on Roche Lightcycler 480 system with qPCR SYBR Green Master Mix (Yeasen, China) according to the manufacturers' instructions. The primer sequences for qRT-PCR are listed in Table 1. Conditions of real-time PCR were as follows: One cycle at 95°C, 5 min; 40 cycles of denaturation (95°C, 10 s), annealing (60°C, 20 s), and extension (72°C, 20 s) with subsequent cooling (4°C, ∞). *β*-Actin was used as the internal control gene. Data were analyzed by using the comparative CT method (2^−∆∆CT^) and expressed as fold changes respective to control.

### 2.10. Western Blot Analysis

Total proteins were extracted with RIPA lysis buffer (Beyotime, China) supplemented with 1% protease inhibitor cocktail (Cwbio, China) and 1% phosphatase inhibitors (Cwbio, China). Nuclear and cytoplasmic proteins were extracted using Nuclear and Cytoplasmic Protein Extraction Kit (Beyotime, China) according to the manufacturer's protocols. Protein concentration was quantified with a BCA protein assay kit following the manufacturer's instructions. Protein samples (30 *μ*g) were then resolved on a 10% SDS-PAGE gel and transferred to a polyvinylidene difluoride membrane. Nonspecific binding sites were blocked by incubating the membranes in bovine serum albumin (BSA) for 1 hour at room temperature. Then, the membranes were incubated overnight at 4°C with primary antibodies against the following: COL1A1 (Affinity, USA, 1 : 1000), ALP (Affinity, USA, 1 : 1000), RUNX2 (Cell Signaling Technology, USA, 1 : 1000), OPN (Affinity, USA, 1 : 1000), OCN (Affinity, USA, 1 : 1000), *β*-CATENIN (Cell Signaling Technology, USA, 1 : 1000), p-AKT (Cell Signaling Technology, USA, 1 : 1000), AKT (Cell Signaling Technology, USA, 1 : 1000), p-GSK-3*β* (Cell Signaling Technology, USA, 1 : 1000), GSK-3*β* (Cell Signaling Technology, USA, 1 : 1000), LAMIN B1 (Proteintech, China, 1 : 5000), and *β*-actin (Yeasen, China, 1 : 2000). After washing, the membranes were incubated with horseradish peroxidase-conjugated secondary antibody (Beyotime, China, 1 : 2000) for 1 hour at room temperature, and the immunoreaction signals were detected with the ECL reagent (Millipore, USA). For quantification, the intensity of each band was measured by the Image J software.

### 2.11. Immunofluorescence Staining for *β*-Catenin Nuclear Translocation

Cells were seeded in confocal dishes at a density of 2 × 10^4^/cm^2^ and cultured for 24 h. The cells were washed with cold PBS three times, fixed, and permeabilized with ice-cold methanol for 5 min. The cells were then washed with PBS three times and blocked with 5% of BSA in PBST for 1 hour at room temperature. Samples were then incubated with rabbit monoclonal anti-*β*-CATENIN antibody (Cell Signaling Technology, USA, 1 : 200) overnight at 4°C followed by incubation with Alexa Fluor 594 Donkey Anti-Rabbit IgG secondary antibody (Yeasen, China, 1 : 500). DAPI (Beyotime, China, 1 : 500) was used to counterstain cell nuclei for 5 min at room temperature. Finally, fluorescence was visualized using confocal microscopy (Carl Zeiss, Germany).

### 2.12. Statistical Analysis

All data were presented as the mean ± standard deviation (SD) of three independent experiments. Statistical difference was analyzed via one-way ANOVA. A *p* value < 0.05 was considered statistically significant. The SPSS 20.0 software (IBM Corp, USA) was used for all data analysis.

## 3. Results

### 3.1. Characterization of rBMSCs

**Figure 1:**
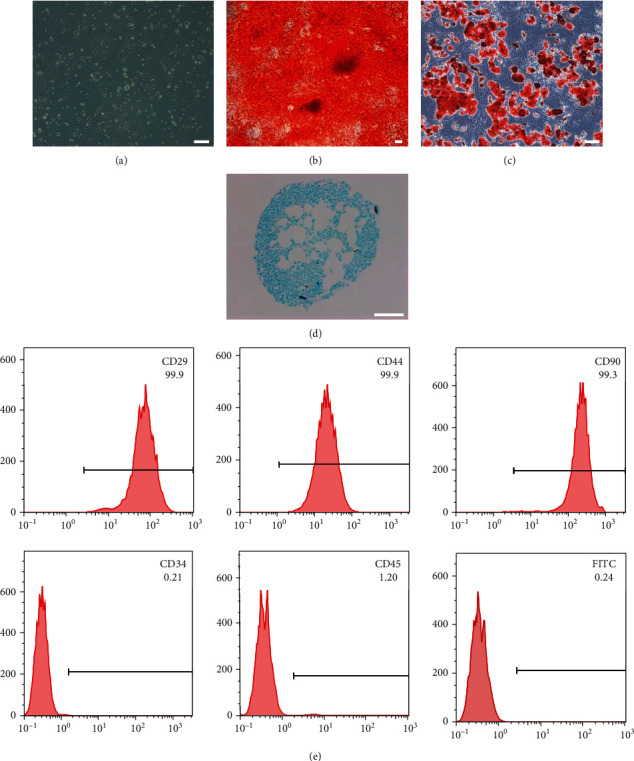
Characterization of rBMSCs. (a) Primary culture of rBMSCs. (b–d) Multiple differentiation ability of rBMSCs. Cells were treated with osteogenic induction medium for 14 days and stained with (b) Alizarin Red. Cells were treated with adipogenic induction medium for 14 days and stained with (c) Oil Red “O.” Cells were treated with chondrogenic induction medium for 21 days and stained with (d) Alcian blue. (e) Flow cytometry analysis of surface markers of rBMSCs. rBMSCs were positive for mesenchymal stem cell markers (CD29, CD44, and CD90), but negative for hematopoietic cell markers (CD34 and CD45). The FITC-conjugated mouse IgG isotype was used as a negative control. Scale bar, 100 *μ*m.

As shown in Figure 1(a), rBMSCs exhibited adherent growth with polygon or fusiform morphology. To confirm multiple differentiation ability, cells were cultured in osteogenic induction medium, adipogenic induction medium, or chondrogenic induction medium. Cells were able to be differentiated into osteoblasts, adipocytes, and chondrocytes as determined by Alizarin Red staining, Oil Red “O” staining, and Alcian blue staining, respectively (Figures 1(b)–1(d)). Moreover, flow cytometric characterization analysis showed that the cells expressed high levels of mesenchymal stem cell markers CD29 (99.9%), CD44 (99.9%), and CD90 (99.3%), but low levels of negative markers CD34 (0.21%) and CD45 (1.2%) (Figure 1(e)). Taken together, these results indicate that the isolated rBMSCs are stem cells of mesenchymal origin with multilineage differentiation potential.

### 3.2. DA Does Not Interfere with the Apoptosis and Proliferation of rBMSCs

**Figure 2:**
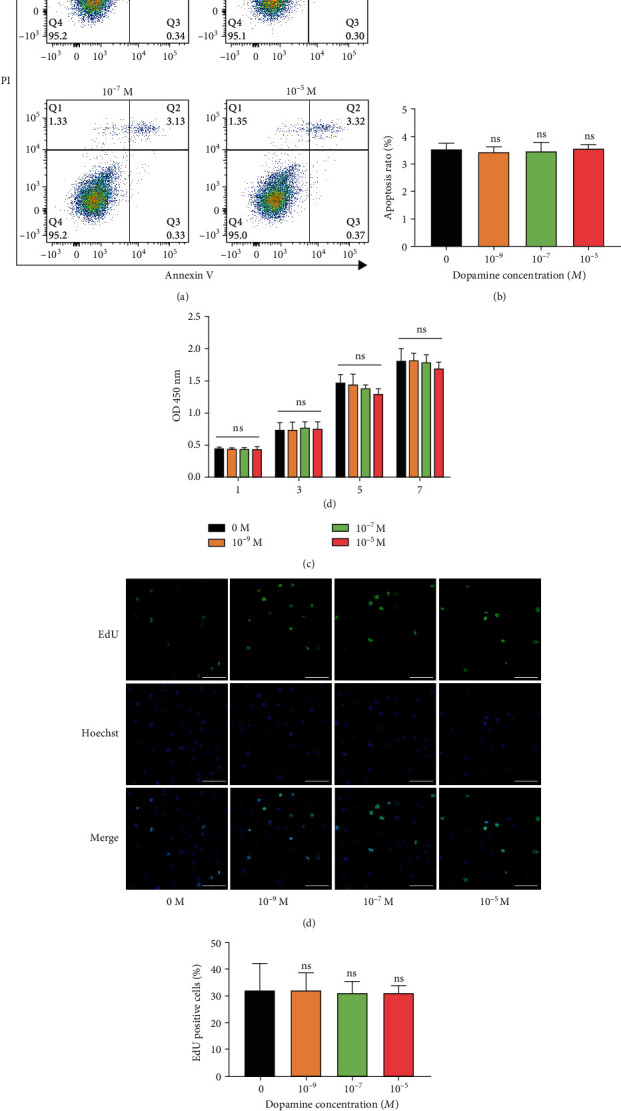
DA does not influence the apoptosis and proliferation of rBMSCs. (a) rBMSCs were treated with different concentrations of DA for 2 days. Flow cytometric analysis of apoptosis using Annexin V-FITC/PI staining. (b) Apoptosis rate analysis. (c) Effect of DA on the proliferation of rBMSCs for 1, 3, 5, and 7 days evaluated by CCK-8 assay. (d) Effect of DA on the proliferation of rBMSCs for 1 day evaluated by EdU assay. Proliferating nuclei were labeled with EdU (green), and the nuclei of all cells were labeled with Hoechst (blue). (e) The cell proliferation ratio of the indicated groups was expressed as the ratio of EdU-positive cells to total Hoechst-positive cells. Scale bar, 100 *μ*m. ns: no significance.

To elucidate the effect of DA on rBMSC apoptosis, the cells were treated with DA at a series of concentrations (0, 10^−9^, 10^−7^, and 10^−5^ M) for 2 days and then harvested for Annexin V/PI staining. Q1 (Annexin V–/PI+) represents necrotic cells, Q2 (Annexin V+/PI+) represents late apoptotic cells, Q3 (Annexin V+/PI–) represents early apoptotic cells, and Q4 (Annexin V–/PI–) represents live cells. The apoptosis rate was calculated as the percentage of early and late apoptotic cells. No significant difference in apoptosis rate was observed among these concentrations (0, 10^−9^, 10^−7^, and 10^−5^ M) (Figures 2(a) and 2(b)). Furthermore, CCK-8 assay was conducted to detect the effect of DA on the proliferation of rBMSCs. No statistically significant difference was found among these concentrations (0, 10^−9^, 10^−7^, and 10^−5^ M) for 1, 3, 5, and 7 days (Figure 2(c)). Consistent with CCK-8 results, EdU assay also showed that DA did not interfere with the ratio of EdU-positive cells (Figures 2(d) and 2(e)). The above results reveal that DA at a concentration of 10^−5^ M or less has no toxic effect on rBMSCs.

### 3.3. DA Suppresses Osteogenic Differentiation and Promotes Adipogenic Differentiation of rBMSCs

**Figure 3:**
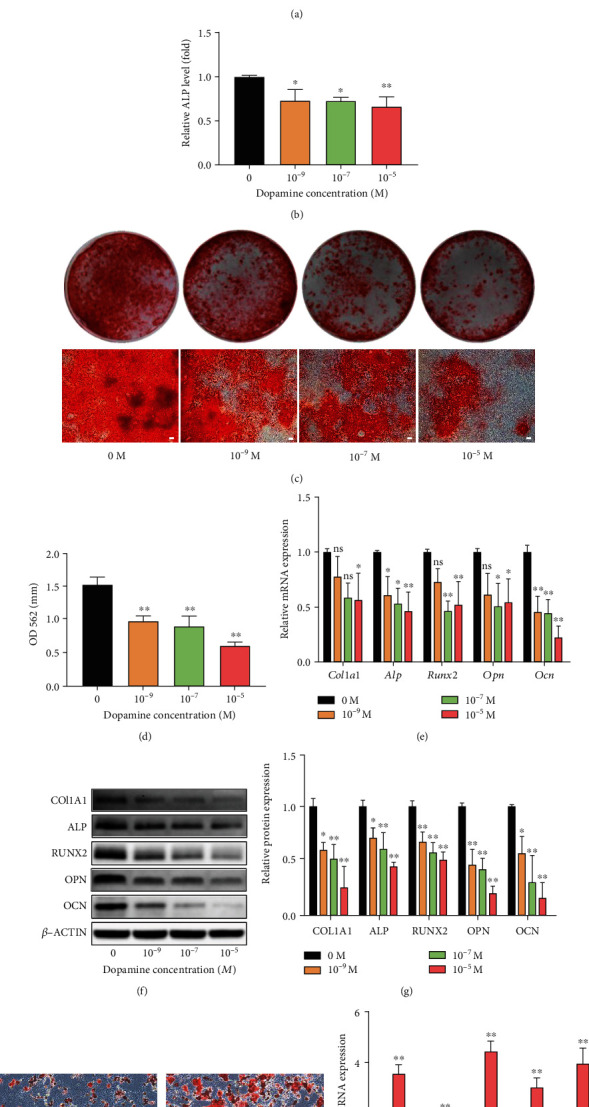
DA inhibits osteogenic differentiation and promotes adipogenic differentiation of rBMSCs. (a) ALP staining of rBMSCs treated with different concentrations of DA after 7 days of differentiation. Optical photos (upper photo) and microscopic images (lower photo). (b) ALP activity of rBMSCs treated with different concentrations of DA after 7 days of differentiation. (c) Calcium nodules assessed by Alizarin Red staining after 14-day osteogenic induction. Optical photos (upper photo) and microscopic images (lower photo). (d) Semiquantification of Alizarin Red staining. (e) The effect of DA on osteogenic-related mRNA expression in rBMSCs evaluated by qRT-PCR assay after 7 days of differentiation. (f, g) The expression levels of osteogenic-related proteins (COL1A1, ALP, RUNX2, OPN, and OCN) detected by western blot. (f) and quantified (g) in the indicated groups with 7-day osteogenic induction. (h) Oil Red “O” staining of rBMSCs treated with different concentrations of DA after 14-day adipogenic induction. (i) The effect of DA on adipogenic-related mRNA (*Adipoq*, *Cebp*, *Fabp4*, *Pparr*, and *Plin1*) expression in rBMSCs evaluated by qRT-PCR assay after 14-day adipogenic induction. Scale bar, 100 *μ*m. ^∗^*p* < 0.05 and ^∗∗^*p* < 0.01.

To investigate the role of DA in osteogenic differentiation, we firstly measured ALP staining, ALP activity, and mineralized nodule formation in rBMSCs with or without DA (10^−9^, 10^−7^, and 10^−5^ M) treatment. ALP staining and ALP activity were significantly suppressed by DA after 7 days of differentiation (Figures 3(a) and 3(b)). Additionally, Alizarin Red staining revealed that after 14-day osteogenic induction, DA markedly suppressed mineralized nodule formation in rBMSCs (Figures 3(c) and 3(d)). Furthermore, we assessed the expression of specific osteogenic markers (*Col1a1*, *Alp*, *Runx2*, *Opn*, and *Ocn)* by qRT-PCR and levels of the corresponding proteins by western blot. Results showed that during osteogenic differentiation of rBMSCs, both mRNA and protein levels of these osteogenic marker genes were significantly reduced upon treatment with DA (Figures 3(e)–3(g)). Besides, to investigate the role of DA in adipogenic differentiation, we performed Oil Red “O” staining and qRT-PCR in rBMSCs with or without DA (10^−5^ M) treatment. Results showed that DA markedly suppressed lipid droplet formation and mRNA levels of adipogenic marker genes (*Adipoq*, *Cebp*, *Fabp4*, *Pparr*, and *Plin1*) in rBMSCs after 14-day induction (Figures 3(h) and 3(i)). Taken together, these results indicate that DA suppresses osteogenic differentiation and promotes adipogenic differentiation of rBMSCs.

### 3.4. DA Suppresses AKT/GSK-3*β*/*β*-Catenin Signaling Pathway during Osteogenic Differentiation of rBMSCs

**Figure 4:**
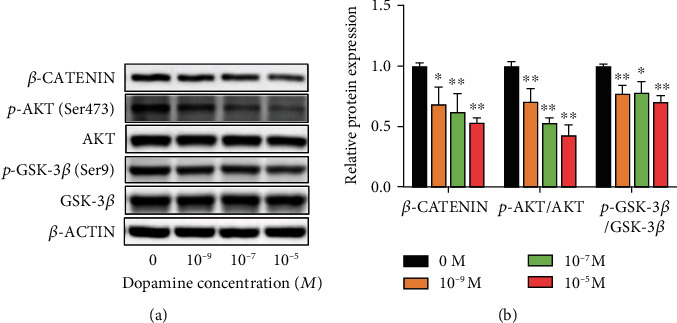
DA suppresses AKT/GSK-3*β*/*β*-catenin signaling pathway during osteogenic differentiation of rBMSCs. (a) Expression levels of AKT, p-AKT, GSK-3*β*, p-GSK-3*β*, and *β*-CATENIN analyzed by western blot assay after 24 h of differentiation. (b) Quantification of p-AKT/AKT ratio, p-GSK-3*β*/GSK-3*β* ratio, and *β*-catenin. ^∗^*p* < 0.05 and ^∗∗^*p* < 0.01.

To assess whether DA suppresses osteogenic differentiation of rBMSCs via the AKT/GSK-3*β*/*β*-catenin signaling pathway, we carried out western blot to detect the protein expression of AKT, phosphorylated AKT (p-AKT), GSK-3*β*, phosphorylated GSK-3*β* (p-GSK-3*β*), and *β*-catenin in rBMSCs in response to treatment with DA (0, 10^−9^, 10^−7^, and 10^−5^ M). We found that the ratio of p-AKT/AKT, p-GSK-3*β*/GSK-3*β*, and *β*-CATENIN was reduced at 24 h after DA treatment. However, no significant change was observed in the total protein expression of AKT and GSK-3*β* (Figures 4(a) and 4(b)). These results indicate that DA suppresses AKT/GSK-3*β*/*β*-catenin signaling pathway during osteogenic differentiation of rBMSCs.

### 3.5. LiCl Rescues the Inhibitory Effects of DA on Osteogenic Differentiation of rBMSCs

**Figure 5:**
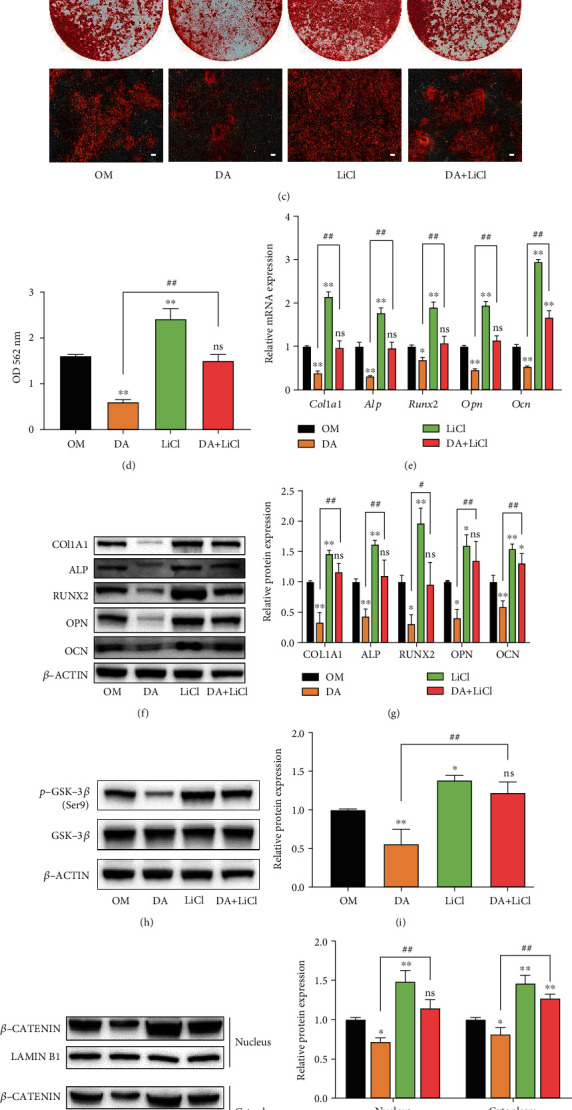
LiCl rescues the attenuation of DA on rBMSC osteogenic differentiation via the AKT/GSK-3*β*/*β*-catenin pathway. rBMSCs were pretreated with 5 mM LiCl for 2 h and then cultured in osteogenic medium containing 10^−5^ M DA. (a) ALP staining of rBMSCs after 7 days of differentiation. Optical photos (upper photo) and microscopic images (lower photo). (b) ALP activity of rBMSCs measured after 7 days of differentiation. (c) Calcium nodules assessed by Alizarin Red staining after 14 days of differentiation. Optical photos (upper photo) and microscopic images (lower photo). (d) Relative quantification of Alizarin Red staining. (e) Expressions of *Col1a1*, *Alp*, *Runx2*, *Opn*, and *Ocn* measured by qRT-PCR assay after 7 days of differentiation. (f, g) Expression of COL1A1, ALP, RUNX2, OPN, and OCN measured by western blot assay after 7 days of differentiation. (h) Expression of GSK-3*β* and p-GSK-3*β* measured by western blot assay after 24 h of differentiation. (i) Quantification of p-GSK-3*β*/GSK-3*β* ratio. (j, k) The expression levels of nuclear and cytoplasmic *β*-CATENIN analyzed by western blot. (j) and quantified (k) in the indicated groups after 24 h of differentiation. (l) The translocation of *β*-CATENIN in the nucleus assessed by immunofluorescence assay after 24 h of differentiation. *β*-CATENIN was stained as red and nuclei were stained by DAPI showing blue. Scale bar, 100 *μ*m. ^∗^*p* < 0.05 and ^∗∗^*p* < 0.01 compared to OM group; ^#^*p* < 0.05 and ^##^*p* < 0.01 compared to DA group; ns: no significance.

To determine whether AKT/GSK-3*β*/*β*-catenin signaling pathway plays a role in the inhibitory effects of DA on osteogenic differentiation of rBMSCs, rBMSCs were pretreated with or without 5 mM LiCl (GSK-3*β* inhibitor) for 2 h. Interestingly, we found LiCl treatment rescued the inhibitory effects of DA (10^−5^ M) on ALP staining, ALP activity, and mineralized nodule formation in rBMSCs (Figures 5(a)–5(d)). Moreover, the inhibitory effects of DA on the expression of osteogenic-related genes (*Col1a1*, *Alp*, *Runx2*, *Opn*, and *Ocn*) and proteins (COL1A1, ALP, RUNX2, OPN, and OCN) were also rescued by LiCl treatment (Figures 5(e)–5(g)). Then, we conducted western blot to analyze the effect of LiCl on AKT/GSK-3*β*/*β*-catenin signaling pathway. It was found that LiCl rescued the inhibitory effects of DA on p-GSK-3*β*/GSK-3*β* and *β*-CATENIN protein expression (Figures 5(h)–5(k)). Furthermore, we confirmed the above results using cellular immunofluorescence assay. Immunofluorescence assays showed that DA treatment inhibited the translocation of *β*-catenin into the nucleus. After pretreatment with LiCl, more *β*-catenin was translocated into the nucleus compared to the group treated with DA alone (Figure 5(l)). Collectively, these results indicate that the GSK-3*β* inhibitor LiCl rescues the inhibitory effects of DA on osteogenic differentiation of rBMSCs.

### 3.6. LY294002 Exacerbates the Inhibitory Effects of DA on Osteogenic Differentiation of rBMSCs

**Figure 6:**
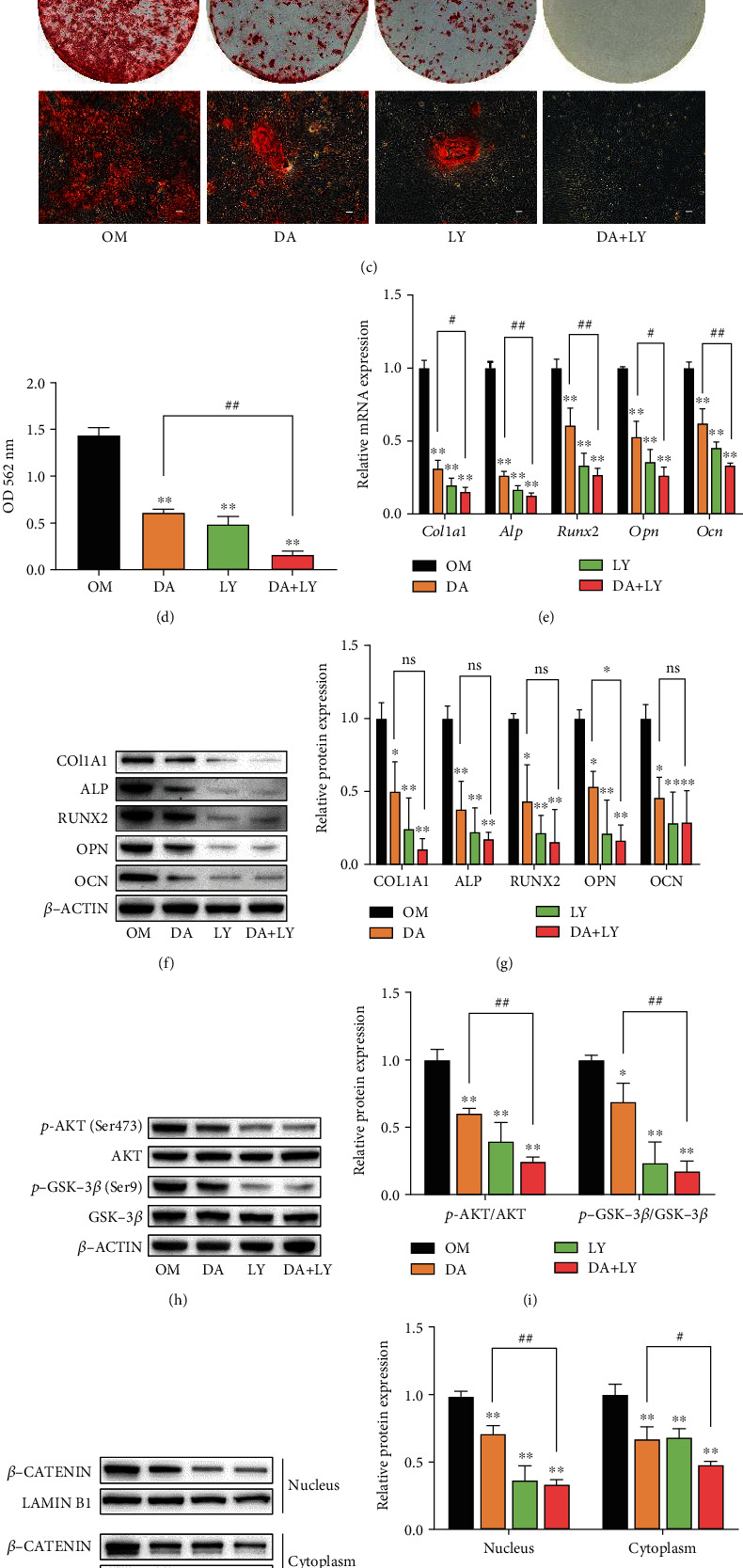
LY294002 exacerbates the attenuation of DA on rBMSC osteogenic differentiation via the AKT/GSK-3*β*/*β*-catenin pathway. rBMSCs were pretreated with 5 *μ*M LY294002 for 2 h and then cultured in osteogenic medium containing 10^−5^ M DA. (a) ALP staining of rBMSCs after 7 days of differentiation. Optical photos (upper photo) and microscopic images (lower photo). (b) ALP activity of rBMSCs measured after 7 days of differentiation. (c) Calcium nodules assessed by Alizarin Red staining after 14 days of differentiation. Optical photos (upper photo) and microscopic images (lower photo). (d) Relative quantification of Alizarin Red staining. (e) Expressions of *Col1a1*, *Alp*, *Runx2*, *Opn*, and *Ocn* measured by qRT-PCR assay after 7 days of differentiation. (f, g) Expression of COL1A1, ALP, RUNX2, OPN, and OCN measured by western blot assay after 7 days of differentiation. (h) Expression of GSK-3*β* and p-GSK-3*β* measured by western blot assay after 24 h of differentiation. (i) Quantification of p-GSK-3*β*/GSK-3*β* ratio. (j, k) The expression levels of nuclear and cytoplasmic *β*-CATENIN analyzed by western blot. (j) and quantified (k) in the indicated groups after 24 h of differentiation. (l) The translocation of *β*-CATENIN in the nucleus assessed by immunofluorescence assay after 24 h of differentiation. *β*-CATENIN was stained as red, and nuclei were stained by DAPI showing blue. Scale bar, 100 *μ*m. ^∗^*p* < 0.05 and ^∗∗^*p* < 0.01 compared to OM group; ^#^*p* < 0.05 and ^##^*p* < 0.01 compared to DA group; ns: no significance.

To further investigate whether AKT/GSK-3*β*/*β*-catenin signaling pathway is involved in the inhibitory effects of DA on osteogenic differentiation of rBMSCs, rBMSCs were pretreated with or without 5 *μ*M LY294002 (AKT inhibitor) for 2 h. The results showed that LY294002 administration exacerbated the inhibitory effects of DA (10^−5^ M) on ALP staining, ALP activity, and mineralized nodule formation in rBMSCs (Figures 6(a)–6(d)). Moreover, LY294002 treatment also aggravated the inhibitory effects of DA on the mRNA and protein levels of osteogenic-related genes (*Col1a1*, *Alp*, *Runx2*, *Opn*, and *Ocn*) (Figures 6(e)–6(g)). Furthermore, western blot was conducted to analyze how LY294002 influenced the AKT/GSK-3*β*/*β*-catenin signaling pathway. It was shown that LY294002 exacerbated the inhibitory effects of DA on p-GSK-3*β*/GSK-3*β* and *β*-catenin protein expression (Figures 6(h)–6(k)). We also used cellular immunofluorescence assay to confirm the above result and found that DA treatment inhibited the translocation of *β*-catenin into the nucleus. After pretreatment with LY294002, less *β*-catenin translocated into the nucleus compared to the group treated with DA alone (Figure 6(l)). These findings suggest that the AKT inhibitor LY294002 exacerbates the inhibitory effects of DA on osteogenic differentiation of rBMSCs.

## 4. Discussion

Peripheral DA plays a crucial role in a variety of physiological processes, including sympathetic regulation, hormone secretion, immune activation, blood pressure regulation, renal function, respiration, and gastrointestinal motility [24]. Peripheral DA can be released from a number of different sources, including the adrenal medulla, sympathetic nerves, and other peripheral organs, where DA can function as a regulator of local organs in an autocrine and/or paracrine manner [25]. Previous research has demonstrated that the concentration of DA in bone marrow could range from nanomolar to micromolar [26–28].

DA primarily exerts its function by activation of DA receptors that belong to the G protein-coupled receptor superfamily. DA receptors are divided into two classes: D1-like receptors (D1R and D5R) and D2-like receptors (D2R, D3R, and D4R). The activation of D1-like receptors stimulates adenylate cyclase and subsequently increases the concentration of intracellular cyclic adenosine monophosphate (cAMP), while the activation of D2-like receptors exerts an opposite effect [29, 30]. It has been confirmed that MC3T3-E1 preosteoblast cell line, rat osteoblasts, rBMSCs, and human BMSCs express both D1-like and D2-like receptors, indicating that DA might play a role in osteogenic differentiation [14, 15, 31].

In the current study, we demonstrated that 10^−9^, 10^−7^, and 10^−5^ M DA did not interfere with the apoptosis or proliferation of rBMSCs and significantly suppressed ALP staining and ALP activity in rBMSCs, which is known to be an early differentiation marker of osteogenesis. In the early stages of mineralization, ALP increases hydroxyapatite synthesis by producing inorganic phosphate, subsequently enhancing bone formation and mineralization [32]. In addition, we also found that DA significantly inhibited mineralized nodule formation of rBMSCs, which is known to be a late differentiation marker of osteogenesis. Furthermore, we confirmed that DA inhibited osteogenic differentiation by evaluating the mRNA and protein levels of osteogenesis-related genes and proteins (COL1A1, ALP, RUNX2, OPN, and OCN). Our results are in agreement with some previous studies. It was reported that DA at 10^−9^ and 10^−7^ M suppressed osteoblast mineralization of preosteoblast cell line MC3T3-E1 [17]. A higher concentration (5 × 10^−5^ M) of DA has also been reported to inhibit osteogenic differentiation of BMSCs. However, in the same study, 5 × 10^−9^ M DA had an opposite effect on osteogenic differentiation [14]. Another study also showed that 5 × 10^−5^ M DA could promote osteoblast mineralization in MC3T3-E1 [15]. Such discrepancies might be due to a number of reasons. First, DA is known to have a higher affinity for D2-class receptors than D1-class receptors [33, 34]. At low concentrations, DA could exclusively activate D2-class receptors, while it was able to activate D1-class receptors at high concentrations. In addition, at even higher concentrations, DA could activate D2-class receptors because of a shift of preference to D2-class receptors [33, 35]. As D1-class and D2-class receptors exert opposite effects, different concentrations of DA might have different impacts on osteogenic differentiation. Second, different types of cells were used in different studies. The number of DA receptors could vary between different cell types. In this study, we used primary cultured BMSCs instead of cell lines, which provide a model more closely resembling the bone tissue. Third, DA might activate or inactivate different signaling pathways that lead to opposite effects. DA has been shown to exert biological effects through various signaling pathways including cAMP/PKA pathway, AKT/GSK-3*β* pathway, Wnt/*β*-catenin pathway, and ERK pathway [36–39]. It was found that DA could enhance the osteogenic differentiation of BMSCs by modulating the ERK signaling pathway [14], whereas our results showed that DA suppressed the osteogenic differentiation of rBMSCs by modulating the AKT/GSK-3*β*/*β*-catenin signaling pathway.

**Figure 7:**
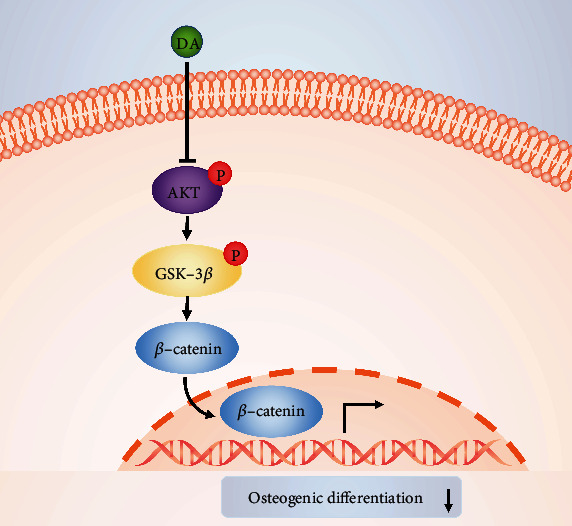
Schematic diagram of DA-mediated osteogenic differentiation by modulating AKT/GSK-3*β*/*β*-catenin pathway in rBMSCs. Intracellular p-AKT and p-GSK-3*β* level was downregulated by DA. Subsequently, translocation of *β*-catenin from the cytoplasm to the nucleus was suppressed, resulting in the inhibition of osteogenic differentiation.

AKT is a serine/threonine-specific protein kinase that plays a key role in many cellular processes, such as cell growth, survival, proliferation, apoptosis, differentiation, migration, transcription, and glucose metabolism [40, 41]. The maximal activation of AKT requires phosphorylation of Ser473 site in the carboxyl-terminal hydrophobic motif [42, 43]. Therefore, in the present study, we chose AKT Ser473 phosphorylation as a marker of AKT activation. GSK-3*β*, the best-characterized downstream target of AKT, is a pleiotropic serine/threonine protein kinase. Activated AKT further phosphorylates GSK-3*β* at Ser9 site, which leads to the inactivation of GSK-3*β* [44]. GSK-3*β* is involved in various intracellular signaling pathways, the most famous of which is Wnt/*β*-catenin signaling pathway. Studies have shown that GSK-3*β* is the connection between PI3K/AKT signaling pathway and Wnt/*β*-catenin signaling pathway, which is crucial to the stability of *β*-catenin in the cytoplasm [45, 46]. As a key negative regulator of *β*-catenin in Wnt/*β*-catenin signaling pathway, GSK-3*β* can promote the degradation of *β*-catenin [47]. Previous researches have demonstrated that AKT/GSK-3*β*/*β*-catenin signaling pathway is involved in osteogenic differentiation and bone regeneration [48–51]. Therefore, we hypothesized that the mechanisms underlying the effect of DA on rBMSCs osteogenic differentiation might be mediated through regulation of the AKT/GSK-3*β*/*β*-catenin signaling pathway. Our results showed that DA downregulated the AKT phosphorylation, GSK-3*β* phosphorylation, and *β*-catenin expression levels. To further verify whether the AKT/GSK-3*β*/*β*-catenin signaling pathway was indeed involved in regulating the osteogenic potential of rBMSCs, AKT inhibitor LY294002 and GSK-3*β* inhibitor LiCl were utilized in the present study. Our results showed that treatment with LiCl rescued the inhibitory effects of DA on osteogenic differentiation of rBMSCs, while LY294002 exacerbated the inhibitory effects of DA on osteogenic differentiation of rBMSCs, which indicated that dopamine suppresses osteogenic differentiation of rat bone marrow-derived mesenchymal stem cells via AKT/GSK-3*β*/*β*-catenin signaling pathway (Figure 7). Besides, the results of Oil Red “O” staining and expression of the adipogenic marker genes (*Adipoq*, *C/ebpα*, *Fabp4*, *Pparγ*, and *Plin1*) showed that 5 × 10^−5^ M DA significantly promoted adipogenic differentiation of rBMSCs. However, the mechanisms of DA on adipogenic differentiation of rBMSCs require further investigation.

Nonetheless, several limitations in our study should be noted to provide ideas for further research. First, we have not investigated which receptor is involved in the effect of DA on osteogenic differentiation. Second, as we did not conduct *in vivo* studies to support our conclusions, there is a need for further animal studies to investigate the effect of DA on bone remodeling. Further studies are required to fully elucidate the underlying mechanisms.

## 5. Conclusions

In conclusion, our study demonstrates that DA suppresses osteogenic differentiation of rBMSCs and that AKT/GSK-3*β*/*β*-catenin signaling pathway is involved in this process. Therefore, our study may provide new insights for understanding the relationship between neurotransmitters and bone remodeling.

## Figures and Tables

**Table 1:** Primer sequences used in quantitative real-time reverse transcription polymerase chain reaction.

Gene Target	Sequence
*Col1a1*	Forward: 5′-TGTTGGTCCTGCTGGCAAGAATG-3′
	Reverse: 5′-GTCACCTTGTTCGCCTGTCTCAC-3′
*Alp*	Forward: 5′-TATGGCTCACCTGCTTCACGG-3′
	Reverse: 5′-GCTGTCCATTGTGGGCTCTTG-3
*Runx2*	Forward: 5′-TCCGCCACCACTCACTACCAC-3′
	Reverse: 5′-GGAACTGATAGGACGCTGACGAAG-3′
*Opn*	Forward: 5′-CTTTTGCCTGTTCGGCCTTG-3′
	Reverse: 5′-TGAGATGGGTCAGGCTTCAG-3′
*Ocn*	Forward: 5′-AGACTCCGGCGCTACCTCAAC-3′
	Reverse: 5′-GGCGTCCTGGAAGCCAATGTG-3′
*Adipoq*	Forward: 5′-CATTATGACGGCAGCACTGG-3′
	Reverse: 5′-CCTTCCCCATACACTTGGAGC-3′
*C/ebpα*	Forward: 5′-AGTCGGTGGATAAGAACAGCAACG-3′
	Reverse: 5′-CGGTCATTGTCACTGGTCAACTCC-3′
*Fabp4*	Forward: 5′-GTGGTGGAATGTGTCATGAAAG-3′
	Reverse: 5′-TTGATGCAAATTTCAGTCCAGG-3′
*Pparγ*	Forward: 5′-CCATCGAGGACATCCAAGACAACC-3′
	Reverse: 5′-GCTCTGTGACAATCTGCCTGAGG-3′
*Plin1*	Forward: 5′-GGTACACACCGTGCAGAAGA-3′
	Reverse: 5′-GGCATCGGATAGGGACATGG-3′
*β-Actin*	Forward: 5′-TACAACCTTCTTGCAGCTCCTC-3′
	Reverse: 5′-CATACCCACCATCACACCCTG-3′

## Data Availability

The data used to support the findings of this study are available from the corresponding author upon reasonable request.
